# Roles and molecular mechanisms of EMILIN-1 in physiology and disease: a narrative review

**DOI:** 10.3389/fmed.2026.1915687

**Published:** 2026-08-25

**Authors:** Yaoxin Wang, Huan Zang, Yuqing Huang, Zhongfeng Wang, Xiaomei Wang

**Affiliations:** 1 Department of Hepatology, Center of Infectious Diseases and Pathogen Biology, The First Hospital of Jilin University, Changchun, China; 2 Jilin Provincial Key Laboratory of Metabolic Liver Diseases, The First Hospital of Jilin University, Changchun, China; 3 Department of Dermatology, Affiliated Hospital of Changchun University of Traditional Chinese Medicine, Changchun, Jilin, China

**Keywords:** cancer, EMILIN-1, extracellular matrix, inflammation, lymphangiogenesis, TGF-β/Smad signaling, vascular diseases

## Abstract

Elastin microfibril interface located protein 1 (EMILIN-1) is a homotrimeric glycoprotein of the extracellular matrix (ECM) family that participates in ECM organization and regulates cell signaling. It is critical for maintaining tissue homeostasis, intercellular communication, and modulation of pathological microenvironments. Accumulating evidence indicates that the development and progression of various human diseases, including chronic inflammatory diseases, lymphatic diseases, and cancers, are closely associated with abnormal expression or dysfunction of EMILIN-1. Remarkably, the biological effects of EMILIN-1 are highly context dependent and cell type dependent. EMILIN-1 acts as a well-recognized tumor suppressor in malignancies such as head and neck squamous cell carcinoma and breast cancer, yet it promotes lymphatic disease progression through modulation of transforming growth factor β (TGF-β) signaling. This review summarizes the molecular structural characteristics, physiological functions, and major regulatory pathways of EMILIN-1, with particular emphasis on functional heterogeneity and the underlying mechanisms across diverse diseases. We also highlight recent advances in clinical translation, followed by a discussion of current research bottlenecks and prospective directions. These insights establish a theoretical framework for developing diagnostic biomarkers and optimizing targeted therapeutic strategies.

## 1 Introduction

The extracellular matrix (ECM) forms a complex network of cell secreted proteins and polysaccharides that provide structural support and regulate cellular behavior through receptor mediated signaling. The ECM interacts with cell surface receptors to regulate important physiological processes, including cell proliferation, differentiation, and migration. Alterations in ECM composition and organization are tightly correlated with the development and progression of diverse disease contexts. In 1983, Bressan et al. identified a novel 115-kDa matrix glycoprotein isolated from the extracellular matrix of chicken aorta, which was subsequently named elastin microfibril interface located protein 1 (EMILIN-1) (1, 2). Subsequent studies have revealed that EMILIN-1 localizes to extracellular regions surrounding elastic laminae and smooth muscle cells. Biochemical and immunochemical analyses revealed no evidence of significant structural or immunological similarities between EMILIN-1 and known ECM proteins, such as fibronectin and laminin, indicating that EMILIN-1 represents a distinct extracellular matrix component with unique structural and functional properties (3). EMILIN-1 is a homotrimeric glycoprotein serving as a molecular bridge between elastic fibers and microfibrils. Accordingly, it is indispensable for elastic fiber assembly and ECM structural integrity. Beyond its structural role, EMILIN-1 modulates cellular events and tissue homeostasis through EMILIN-1/integrin signaling pathways (4). This dual structural and signaling capacity highlights its biological importance in ECM organization and makes EMILIN-1 an important target for ECM related research.

Early investigations focused on the role of EMILIN-1 in elastic fiber assembly and the maintenance of vascular integrity, whereas recent studies have uncovered its roles in cancer suppression, modulation of inflammatory responses, and tissue repair. Cumulative evidence indicates that abnormal expression or dysfunction of EMILIN-1 is involved in the pathogenesis of cardiovascular diseases, cancer, and autoimmune diseases, highlighting its promising potential for clinical translation.

Nevertheless, several critical questions remain to be addressed, such as the precise molecular mechanisms underlying EMILIN-1 function, its context dependent regulatory roles in different diseases, and the obstacles limiting its clinical translation. Herein, we review recent progress in EMILIN-1 research, focusing on its molecular structure, physiological functions, disease-related regulatory mechanisms, and therapeutic potential. By integrating existing evidence and highlighting existing knowledge gaps, we aim to construct an integrated framework to guide future basic and translational investigations.

## 2 Molecular characteristics and physiological functions of EMILIN-1

### 2.1 Molecular structural features

The EMILIN/Multimerin family represents a highly conserved group of homologous trimeric ECM glycoproteins, consisting of EMILIN-1, EMILIN-2, EMILIN-3, Multimerin-1, and Multimerin-2. All members share a conserved modular architecture comprising an N-terminal EMI domain, a collagen like region, a coiled domain responsible for trimerization, and a C-terminal globular C1q (gC1q) domain. These proteins exhibit dual functions in structural support and signal regulation. Notably, EMILIN-1 localizes at the elastin microfibril interface to maintain elastic fiber assembly, inhibit transforming growth factor β (TGF-β) activation via the EMI domain, and regulate cellular behavior through integrin binding mediated by the gC1q domain. Accordingly, they participate broadly in tissue development and homeostasis, particularly in blood vessels, heart valves, and skin, as well as in pathological processes such as cardiovascular diseases, fibrosis, and cancer angiogenesis (4). Among these family members, EMILIN-1 is considered the prototypical and functionally central protein. The human EMILIN-1 gene is located on chromosome 2p23.3 (5), a locus associated with susceptibility to multiple diseases (6). At the protein level, EMILIN-1 contains a C-terminal gC1q domain that represents its major functional region. This domain mediates homotypic interactions facilitating the assembly of EMILIN-1 into a stable homotrimer, which is essential for ECM organization and function (7). In addition, EMILIN-1 contains an N-terminal cysteine rich region and other conserved structural elements that facilitate interactions with ECM components, including elastin and microfibril associated proteins. The coordinated functions of these domains maintain ECM integrity and provides the molecular basis for preserving tissue architecture (4).

### 2.2 Expression characteristics of EMILIN-1

Under physiological conditions, EMILIN-1 exhibits broad tissue distribution and is widely expressed across multiple organs. It is primarily localized within the ECM of connective tissues and is particularly abundant in elastic fiber rich structures, including the vascular wall, dermis, heart valves, myocardial interstitium, and lung parenchyma (8). At the cellular level, EMILIN-1 is mainly synthesized and secreted by fibroblasts, vascular smooth muscle cells, endothelial cells, cardiac fibroblasts, and pulmonary interstitial cells. Dermal fibroblasts act as major effector cells in connective tissue, producing abundant EMILIN-1 to orchestrate ECM assembly and elastic fiber network formation in the skin (2). Vascular smooth muscle cells produce EMILIN-1, which modulates TGF-β signaling to preserve vascular elasticity, maintain myogenic tone, and sustain blood pressure homeostasis (9). Endothelial cells have also been reported to express EMILIN-1 contributes to structural coupling between the endothelium and elastic laminae. Furthermore, EMILIN-1 secreted by cardiac fibroblasts and pulmonary interstitial cells supports the elastic architecture of heart valves, myocardial interstitium, and lung tissue to accommodate organ specific mechanical demands (10). Collectively, these expression patterns are consistent with EMILIN-1’s core functions in maintaining elastic tissue integrity and vascular function regulation.

### 2.3 Core physiological functions of EMILIN-1

The physiological roles of EMILIN-1 are closely linked to its structural characteristics and expression profile and can be categorized into structural support and homeostatic regulation. As a key molecular linker between elastic fibers and microfibrils, EMILIN-1 fulfills mechanical structural functions. Consequently, it contributes to the maintenance of structural integrity and elasticity in vascular wall, skin, lungs, and other elastic tissues (2). Beyond its mechanical function, EMILIN-1 mediates cell ECM to preserve tissue homeostasis. It modulates critical cellular processes, including cell adhesion, inhibition of excess cell proliferation, and maintenance of vascular tone, partly through the modulation of signaling pathways such as TGF-β signaling (9, 11). EMILIN-1 also maintains lymphatic vessel function by regulating lymphatic endothelial cell morphology and behavior, ensuring vessel integrity and efficient lymphatic drainage (12). In summary, EMILIN-1 acts as an indispensable ECM component integrating structural scaffolding and signal regulatory properties to sustain physiological tissue homeostasis.

## 3 Core regulatory pathways of EMILIN-1

**Figure 1:**
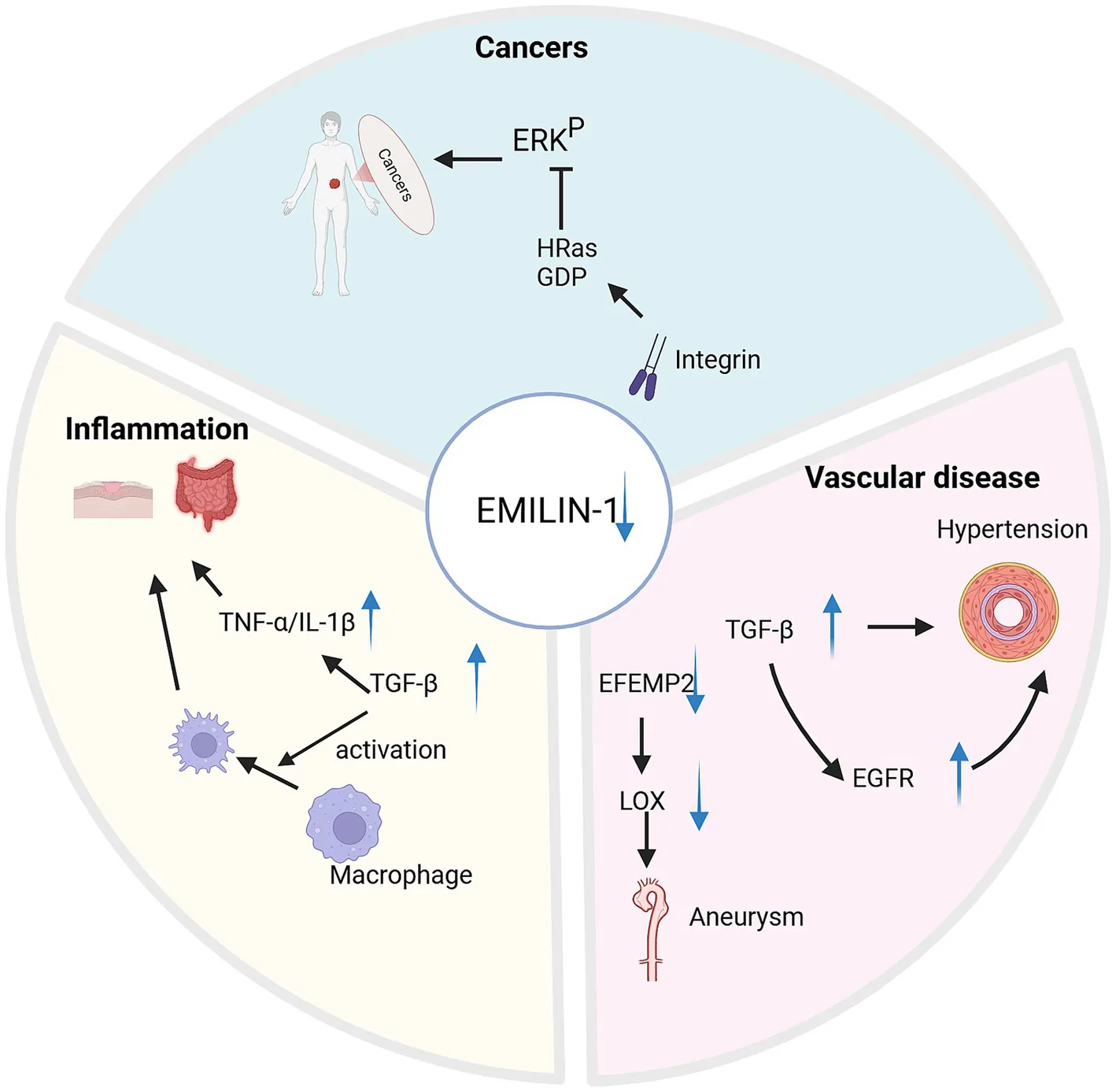
Schematic representation of the role of EMILIN-1 in regulating inflammation, vascular disorders, and cancer progression. EMILIN-1 deficiency contributes to dysregulated immune responses, vascular dysfunction, and tumor development. This schematic was created using BioRender.

EMILIN-1 executes its biological functions through coordinated modulation of multiple signaling axes, forming an interconnected regulatory network. The primary mechanisms mediating EMILIN-1 activity under physiological and pathological contexts include integrin-dependent signaling, the TGF-β/Smad cascade, and pathways governing cell cycle progression and lymphangiogenesis. Together, these pathways regulate diverse biological processes, including cell proliferation, inflammation modulation and extracellular matrix synthesis, and tissue homeostasis maintenance. Importantly, the regulatory effects of EMILIN-1 are highly dependent on the cell type and tissue microenvironment, thus offering a molecular basis for its context specific roles in disease progression (Figure 1).

### 3.1 Integrin mediated signalling pathway

The interaction between EMILIN-1 and integrins is highly selective at the structural level. The central binding interface consists of three Glu933 residues in the disordered loop at the apex of the gC1q trimer that coordinate the Mg2+ ion at the metal ion-dependent adhesion site (MIDAS) of the integrin β1 subunit. This interaction establishes a unique trimer-dimer binding configuration, while residue R904 acts as a cooperative site by forming intrachain salt bridge formation. Importantly, mutation or proteolytic cleavage of Glu933 abrogates integrin binding and consequently prevents activation of downstream signaling pathways (7).

The antiproliferative activity of EMILIN-1 represents a central function in integrin mediated signaling and is mediated through two principal mechanisms. In skin tissue, EMILIN-1 binds to α4β1 and α9β1 integrins expressed on dermal fibroblasts and basal keratinocytes, respectively, thereby upregulating the expression of phosphatase and tensin homolog (PTEN) and suppressing the PI3K/Akt and ERK1/2 pathways. This regulatory mechanism maintains cellular quiescence and preserves tissue homeostasis. Loss of EMILIN-1 reduces PTEN expression and triggers hyperactivation of PI3K/Akt and ERK1/2 signaling, resulting in epidermal and dermal hyperproliferation. Excessive ERK1/2 activation further represses Smad2 signaling, thereby exacerbating homeostatic imbalance in cutaneous tissue (11).

In epithelial and cancer cells, EMILIN-1 engages α4β1 integrin through its gC1q domain, promoting ubiquitination of HRas-GTP and attenuating downstream HRas signaling. Consequently, reduced ERK1/2 phosphorylation suppresses the proliferative RAS–ERK cascade. This effect is highly specific and structurally dependent, as collagen-mediated activation of α2β1 integrin fails to reproduce this response and requires the intact intracellular domain of the α4 integrin subunit (13).

Besides controlling cell proliferation, EMILIN-1 influences the tissue microenvironment through the α4β1 and α9β1 integrins. EMILIN-1 integrin interactions during lymphangiogenesis are crucial for maintaining the structural integrity and drainage function of lymphatic vessels. Disruption of this signaling axis leads to lymphatic dysfunction, lymphedema and an increased risk of lymph node metastasis. These interactions preserve the integrity of the lymphatics in the intestine and promote the resolution of inflammation, limiting the progression of colitis and colorectal cancer. Conversely, impaired EMILIN-1/integrin binding promotes inflammation and susceptibility to cancer development (14).

Moreover, α4β1 integrin mediates EMILIN-1 dependent cell adhesion and migration. EMILIN-1 stimulates fibroblast adhesion and migration and promotes directional migration of placental trophoblasts. These processes involve matrix metalloproteinases, including MT1-MMP and MMP-2. In addition, the Glu933 residue is essential for mediating the adhesion and migration of T cells and endothelial cells (15). Proteolytic processing is an important regulatory step in this pathway. Neutrophil elastase specifically cleaves the region containing Glu933 within the gC1q domain, completely abrogating the EMILIN-1 integrin interactions and its antiproliferative, adhesive and cancer suppressive functions. In contrast, partial cleavage by MMP-3 or MT1-MMP preserves the structural integrity of the gC1q domain, allowing integrin binding and downstream signaling to remain intact (16).

### 3.2 TGF-β signaling pathway

EMILIN-1 is a critical negative regulator of TGF-β signaling. Mechanistically, EMILIN-1 interacts with the pro–TGF-β precursor and inhibits its furin mediated proteolytic processing in the extracellular space, thereby preventing TGF-β maturation and activation (9). Loss of EMILIN-1 abrogates this inhibitory control and results in sustained activation of TGF-β signaling via canonical and non-canonical pathways. Specifically, the Smad dependent pathway, characterized by increased Smad2/3 phosphorylation, is constitutively upregulated even at early stages, whereas MAPK/ERK pathway, reflected by phosphorylated ERK1/2, shows progressive activation with age (17). This regulatory mechanism displays marked cell-type specificity. In vascular smooth muscle cells, EMILIN-1 participates in the regulation of TGF-β signaling and is critical for the preservation of normal myogenic responses to hemodynamic stress and blood pressure (18). In aortic valve tissue, deficiency of EMILIN-1 results in elastic fiber fragmentation, aberrant early angiogenesis and subsequent myofibroblast activation and fibrotic remodeling (17).

The EMILIN-1/TGF-β axis has been demonstrated to be a causal regulator through genetic and pharmacological approaches. TGF-β1 haploinsufficiency is enough to normalize blood pressure in EMILIN-1-deficient mice, while administration of TGF-β neutralizing antibodies reverses hypertension within a short time frame (9). Polymorphisms of the EMILIN-1 gene (e.g., rs2289360 and rs2011616) have been found to be significantly associated with susceptibility to essential hypertension in humans (19, 20). More broadly, the TGF-β/Smad pathway is a key inducer of multiorgan fibrosis affecting the kidney, liver, lung, and heart by inducing fibroblast to myofibroblast differentiation and excessive extracellular matrix deposition (21). Notably, TGF-β simultaneously elevates EMILIN1 expression and aggravates fibrotic lesions in Duchenne muscular dystrophy (DMD) (22).

Besides its profibrotic effects, TGF-β signaling is also crucial for the regulation of apoptosis. This pro-apoptotic function is highly context-dependent, varying according to cell type and microenvironment. TGF-β exerts tumor-suppressive functions during early carcinogenesis but paradoxically promotes cancer cell survival and immune evasion in advanced malignancies (23). Therefore, the dysregulation of the EMILIN-1/TGF-β axis is a critical molecular link between ECM homeostasis, cardiovascular, fibrotic diseases, and cancers.

### 3.3 Cell cycle and lymphangiogenesis regulatory pathways

Besides integrin and TGF-β mediated signaling, EMILIN-1 maintains tissue homeostasis by regulating cell cycle progression and lymphangiogenesis. Regarding cell cycle control, EMILIN-1 exerts antiproliferative activity partially via repressing Aurora kinase signaling. Aurora kinase A is downregulated by EMILIN-1, thereby G0/G1 cell cycle arrest. EMILIN-1 also decreased the protein levels of cell cycle regulators, including CDK1, cyclin B1, cyclin B2, cyclin A2, and cyclin D1, thereby suppressing cell proliferation (24).

EMILIN-1 also governs lymphangiogenesis by sustaining lymphatic endothelial cell structural and functional integrity. Under physiological conditions, it stabilizes intercellular junctions and preserves the normal architecture of lymphatic vessels (12), however, the exact underlying mechanism remains incompletely understood. Conversely, EMILIN-1 deficiency disrupts endothelial junctions, induces morphological abnormalities in lymphatic endothelium, and elevates vascular endothelial growth factor C (VEGF-C) levels, thereby altering lymphatic vessel formation and remodeling (12, 25). Thus, the coordinated regulation of these pathways by EMILIN-1 links cell proliferation control with the preservation of tissue architecture, ultimately contributing to physiological homeostasis.

## 4 Regulatory roles and mechanisms of EMILIN-1 in inflammation, vascular diseases, and cancers

### 4.1 Role and mechanisms of emilin-1 in chronic inflammatory diseases

As an important regulatory glycoprotein in the extracellular matrix, EMILIN-1 plays a crucial anti-inflammatory role during the initiation and progression of chronic inflammatory diseases. These effects are mediated through the three major regulatory mechanisms: the modulation of TGF-β signaling, the regulation of inflammatory cell infiltration, and the maintenance of lymphatic vascular function. Collectively, these mechanisms establish an integrated regulatory network that connects signal transduction, immune responses and tissue homeostasis.

At the level of TGF-β signaling, EMILIN-1 interacts with the latency associated peptide (LAP) of TGF-β through its gC1q domain, thereby restraining excessive TGF-β activation (9). Disruption of this regulatory mechanism, either by loss of EMILIN-1 expression or mutation within its gC1q domain (e.g., E933A), results in dysregulated TGF-β signaling and promotes macrophage polarization toward the pro-inflammatory M1 phenotype, thereby exacerbating tissue inflammation (14). Beyond regulating TGF-β signaling, EMILIN-1 also modulates inflammatory cell infiltration via its gC1q domain by interacting with α4β1/α9β1 integrins expressed on immune cells. This interaction suppresses the adhesion and migration of major inflammatory cells, including neutrophils and monocytes, thereby limiting their excessive accumulation within inflamed tissues. In addition, EMILIN-1 restricts the excessive proliferation of inflammation related cells, thereby attenuating tissue damage. Disruption of the α4β1 integrin binding site eliminates these effects and accelerates the progression of inflammation (14). Furthermore, EMILIN-1 plays a pivotal role in the regulation of lymphatic vascular function. As a structural component of the lymphatic basement membrane, EMILIN-1 preserves endothelial junction integrity and supports efficient lymphatic drainage, facilitating the clearance of inflammatory cells and preventing their prolonged retention in affected tissues. EMILIN-1 deficiency results in impaired lymphatic function despite increased vessel density (e.g., LYVE-1-positive vessels) leading to defective immune cell clearance and exacerbation of chronic inflammation (12, 14).

The anti-inflammatory functions of EMILIN-1 have been demonstrated in multiple experimental models and human disease specimens. In an imiquimod induced psoriasis like dermatitis model, EMILIN-1 deficient mice exhibit enhanced inflammatory infiltration, increased accumulation of Gr-1^+^ neutrophils and F4/80^+^ macrophages, and greater disease severity (26). Consistently, decreased EMILIN-1 has been observed in human psoriatic lesions (11). In the dextran sulfate sodium (DSS) induced colitis model, EMILIN-1 deficiency or defective binding of α4β1 integrin aggravates mucosal injury and promotes inflammatory cell infiltration. Furthermore, EMILIN-1 loss is associated with increased cancer incidence and decreased survival in the Azoxymethane–DSS-induced colorectal cancer model, highlighting its potential role in connecting chronic inflammation with cancer progression (14).

**Figure 2:**
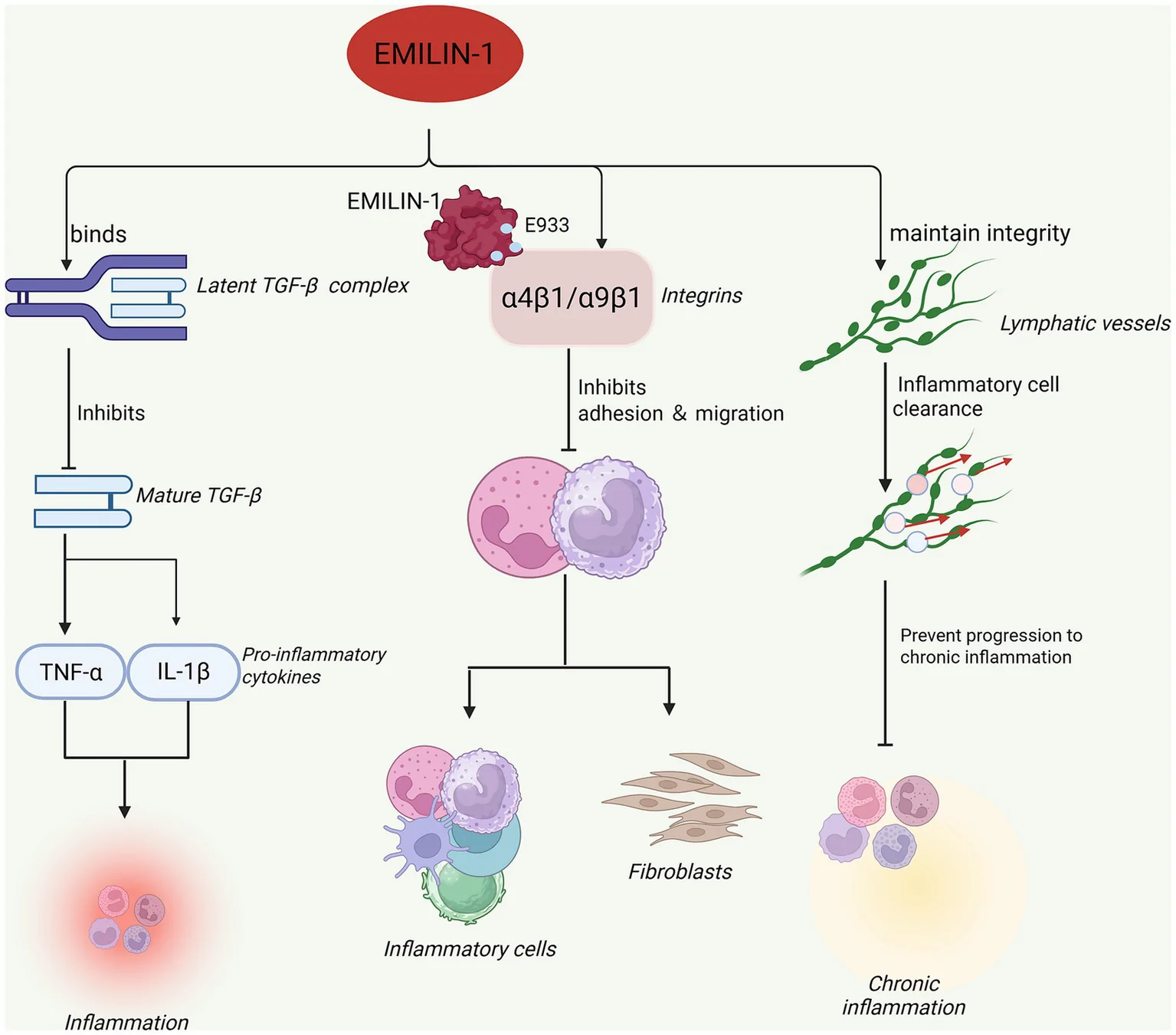
Signaling mechanisms underlying the regulation of inflammatory processes by EMILIN-1. EMILIN-1 deficiency enhances inflammatory responses through disruption of TGF-β signaling, integrin-mediated pathways, and lymphatic vascular function. This figure was created using BioRender.

In summary, EMILIN-1 functions as a critical negative regulator of chronic inflammation by modulating TGF-β signaling, immune cell infiltration, and lymphatic vessel function. The loss or functional impairment of EMILIN-1 disrupts this regulatory network, resulting in excessive inflammatory activation and accelerated disease progression. Therefore, targeting EMILIN-1 associated pathways may represent a promising therapeutic strategy for chronic inflammatory diseases (Figure 2).

### 4.2 Role of EMILIN-1 in vascular system diseases

EMILIN-1 is broadly expressed in arterial and lymphatic tissues and participates in elastic fiber assembly and cell signaling. Dysfunction of EMILIN-1 disrupts vascular and lymphatic homeostasis, primarily through the dysregulation of key signaling pathways, particularly TGF-β signaling. This impairment contributes to the pathogenesis of various vascular and lymphatic disorders, including hypertension, aneurysm, pulmonary arterial hypertension (PAH), and lymphedema. In vascular diseases, EMILIN-1 primarily regulates TGF-β signaling and preserves the structural and functional homeostasis of the vessel wall.

EMILIN-1 is a critical regulator of blood pressure homeostasis. It inhibits TGF-β signaling by interacting with the pro–TGF-β precursor and preventing its maturation and activation. Loss or deficiency of EMILIN-1 increases TGF-β activity, leading to increased vascular resistance, vasoconstriction and hypertension. Notably, this effect is cell type specific, as normalization of blood pressure was only observed after restoration of EMILIN-1 expression in vascular smooth muscle cells, but not in endothelial cells. In addition, pharmacological inhibition of TGF-β signaling can reverse EMILIN-1 deficiency-induced hypertension (9). Mechanistically, EMILIN-1 deficiency further increases myogenic tone in resistance arteries through TGF-β dependent activation of epidermal growth factor receptor (EGFR), a phenomenon reported in experimental models and hypertensive patients (27). Additionally, polymorphisms of EMILIN-1 (e.g., rs3754734 and rs2011616) have also been reported to be associated with an increased susceptibility to hypertension in certain populations (19).

Beyond its role in blood pressure regulation, EMILIN-1 is essential for maintaining vascular structural integrity and regulating vascular remodeling. As a component of the elastic fiber network, EMILIN-1 connects microfibrils with elastin and contributes to elastogenesis and collagen organization. Deficiency of EMILIN-1 impairs EFEMP2 deposition and LOX activity, resulting in defective elastic fiber formation and reduced collagen cross linking. These alterations contribute to arterial tortuosity and increase susceptibility to aortic aneurysm formation (6). Additionally, loss of function mutations in EMILIN-1 have been identified in pediatric patients with severe thoracic aortic aneurysms. Consistent with these observations, experimental studies demonstrate that the absence of EMILIN-1 in vascular smooth muscle cells modulates contractility and reduces proliferative and migratory capacities consistent with aneurysm associated phenotypes. These observations are supported by animal models, in which loss of EMILIN-1 leads to defects in vascular development (28).

EMILIN-1 also participates in pulmonary vascular remodeling associated with PAH. Increased lactate production via lactate dehydrogenase A (LDHA) decreases EMILIN-1 lactylation levels, thereby activating proliferative and profibrotic signaling pathways including YAP/TAZ, Akt–mTOR, and TGF-β1 pathways. These molecular alterations promote pulmonary artery smooth muscle cells proliferation and vascular remodeling. Conversely, inhibition of the LDHA-lactate axis reverses these effects and restores vascular function (29). Furthermore, EMILIN-1 also contributes to vascular development and tissue regeneration, as it is enriched in fetal renal arteries and plays a role in the regulation of endothelial gene expression and regenerative capacity (30).

**Figure 3:**
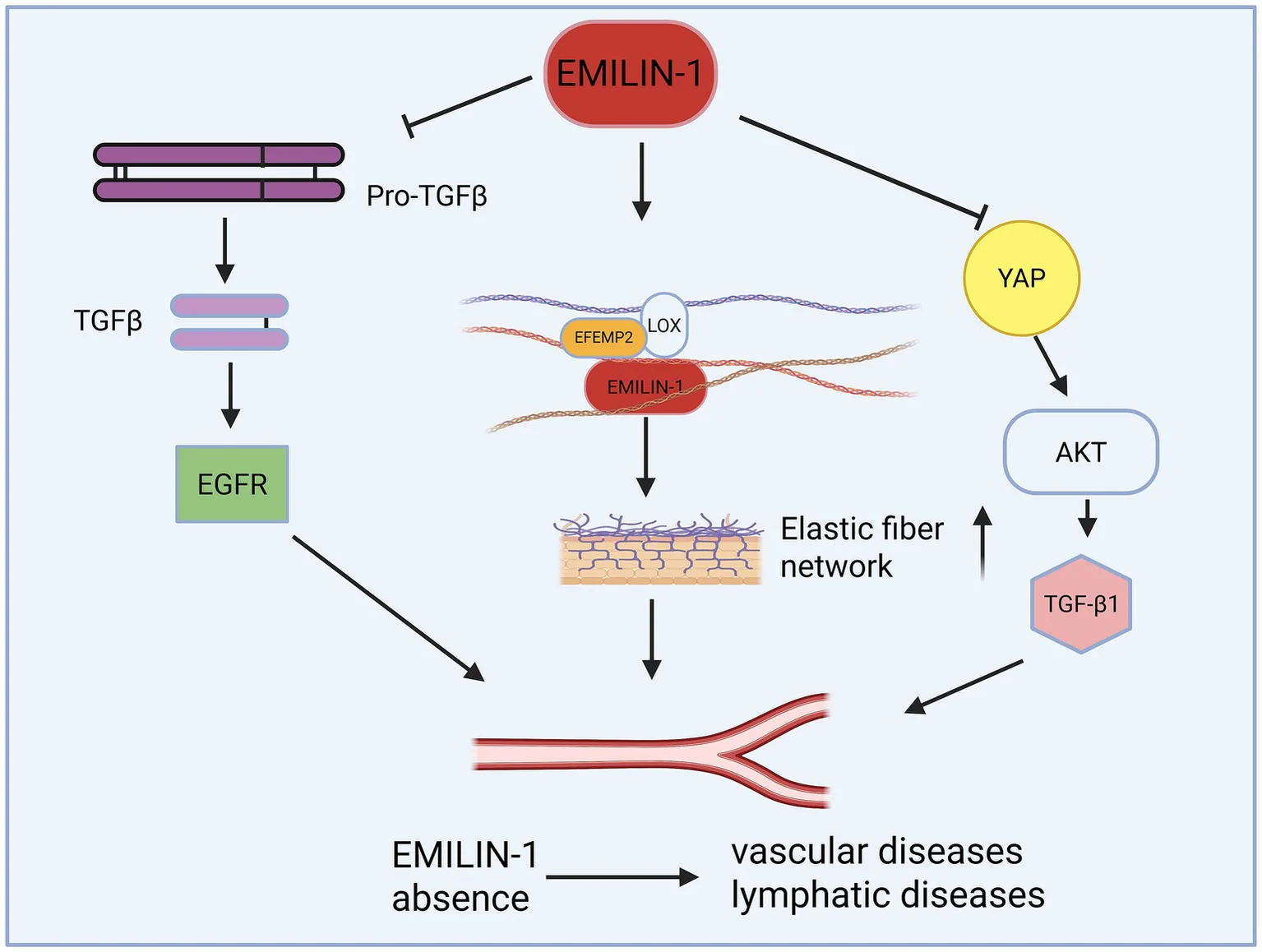
Overview of EMILIN-1-associated signaling pathways involved in vascular diseases. Dysregulation of TGF-β, EFEMP2/LOX, and YAP signaling pathways due to EMILIN-1 deficiency contributes to vascular disease progression. This figure was created using BioRender.

EMILIN-1 is also a major ECM component of the lymphatic system lymphatic system that is essential for maintaining lymphatic vessel integrity and function. It is highly expressed in lymphatic endothelial cells and is a component of anchoring filaments that regulate the stability of lymphatic vessels. EMILIN-1 deficiency results in abnormal lymphatic architecture, reduced anchoring filaments, impaired lymphatic drainage, and mild lymphedema. The findings reveal that ECM protein deficiency can cause lymphatic dysfunction and identify EMILIN-1 as an important regulator of lymphangiogenesis (12). In addition, secondary lymphedema may be induced by EMILIN-1 degradation. Neutrophil elastase cleaves EMILIN-1, leading to disruption of endothelial junction integrity and impaired lymphatic drainage. Pharmacological inhibition of elastase prevents EMILIN-1 degradation, reduces lymphedema and restores lymphatic function. The detection of EMILIN-1 cleavage products in human lymphedema samples further supports its clinical relevance and illuminates a potential ECM targeted therapeutic strategy (Figure 3).

In summary, EMILIN-1 serves as a molecular bridge between vascular and lymphatic homeostasis through its dual functions in structural support and signaling regulation. Dysfunction of EMILIN-1 contributes to the pathogenesis of various vascular and lymphatic disorders. Therapeutic strategies targeting EMILIN-1, such as preventing its degradation, restoring its function or modulating downstream signaling pathways including TGF-β pathway hold promise for the treatment of conditions such as hypertension, aortic aneurysm, PAH, and lymphedema.

### 4.3 Role of EMILIN-1 in cancers

EMILIN-1 exhibits potent tumor suppressive functions by regulating cell signaling and modulating the tumor microenvironment (31). Through interactions with integrins and modulation of oncogenic signaling pathways, EMILIN-1 restrains malignant cell proliferation, invasion, and metastatic dissemination (24).

**Figure 4:**
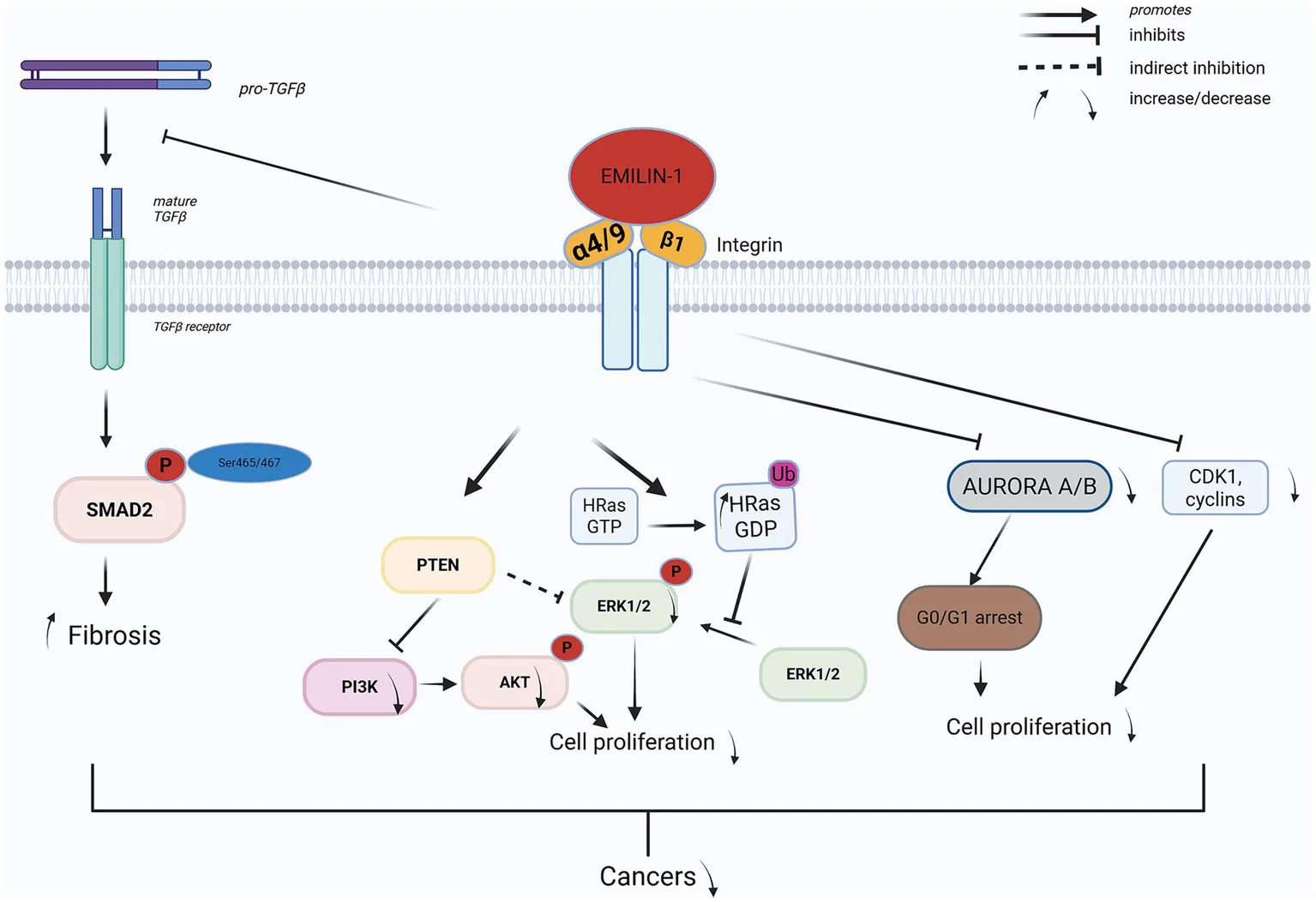
EMILIN-1-associated signaling pathways involved in cancer regulation. Loss of EMILIN-1 results in dysregulation of TGF-β, PTEN, HRas-GTP, and Aurora kinase signaling pathways, thereby promoting tumor progression. This figure was created using BioRender.

At the mechanistic level, the tumor-suppressive activity of EMILIN-1 critically relies on the structural integrity of its gC1q domain. EMILIN-1 interacts with α4β1 integrin to modulate the EMILIN-1/α4β1/HRas-ERK signaling axis, which consequently suppresses cancer cell proliferation (13). This effect requires a functional intracellular domain of the α4 integrin subunit, as disruption of this structure abolishes downstream signaling transduction. Furthermore, EMILIN-1 can be cleaved by neutrophil elastase into functionally inactive fragments, resulting in loss of structural integrity and attenuation of its tumor-suppressive activity in the cancer microenvironment, as shown in sarcoma and ovarian cancer (16). In addition, disruption of the EMILIN-1 and β1 integrin interaction increases inflammatory infiltration and promotes tumorigenesis, at least partially, through impaired lymphatic function and defective inflammatory resolution, particularly in colorectal cancer (14) (Figure 4).

EMILIN-1 exerts context dependent effects across different cancer types, and its reduced expression or epigenetic silencing is frequently associated with more aggressive phenotypes (Table 1). EMILIN-1 and TSPAN9 cooperatively suppress gastric cancer cell migration and invasion, and both are correlated with cancer associated fibroblast infiltration and poor prognosis (32). In colorectal cancer, EMILIN-1 is a downstream target of RORB and the activation of the RORB/EMILIN-1 axis inhibits metastasis, while its disruption promotes tumor dissemination (33). Expression of EMILIN-1 is significantly downregulated in breast cancer tissues and its deficiency accelerates HER2 driven tumorigenesis. Moreover, EMILIN-1 contributes to radiotherapy sensitivity and may serve as a circulating biomarker for predicting treatment response (34–36). EMILIN-1 dysregulation has also been implicated in the progression of multiple other malignancies. In skin cancers, EMILIN-1 deficiency enhances proliferative activity, increases ERK1/2 signaling, and promotes lymphangiogenesis and lymph node metastasis (25). In uterine carcinosarcoma, EMILIN-1 promoter hypermethylation correlates with cancer vascularization (37). In rhabdomyosarcoma, hypermethylation downregulates expression of EMILIN-1, which can be reversed by demethylating agents, suggesting its role as a subtype specific biomarker (38). Furthermore, EMILIN-1 has been identified as a prognostic factor in HPV negative oropharyngeal cancer, a hub gene in hepatitis B virus associated hepatocellular carcinoma, and a stromal regulator in non-small cell lung cancer (39–41). In neuroblastoma, EMILIN-1 is recognized as a core component of the disulfidptosis molecular signature, and *in vitro* functional experiments confirm that EMILIN-1 knockdown promotes tumor cell migration, invasion and colony formation (42).

**Table 1:** Heterogeneous regulatory effects of EMILIN-1 in cancers.

Cancer types	Regulatory mechanisms and functions	References
Gastric cancer	Suppress gastric cancer cell migration and invasion	(32)
Colorectal cancer	Inhibits metastasis	(33)
Breast cancer	Accelerates HER2	(34)
Skin cancers	Increases ERK1/2 signaling	(25)
Uterine carcinosarcoma	Promoter hypermethylation	(37)
Rhabdomyosarcoma	Hypermethylation	(38)
Neuroblastoma	Integrin mediated signaling	(42)

From a clinical perspective, EMILIN-1 substantial broad translational potential in cancer diagnosis, prognosis, and therapy. Its expression level may serve as a potential prognostic biomarker in several cancers and reduced EMILIN-1 expression is frequently associated with unfavorable clinical outcomes. In particular, its epigenetic status, including DNA methylation patterns may facilitate in the classification of cancer subtypes as shown in rhabdomyosarcoma. EMILIN-1 is also a potential therapeutic target, and strategies aimed at restoring EMILIN-1 expression, preventing its proteolytic degradation, or modulating upstream regulatory pathways, such as the RORB/EMILIN-1 axis, have shown therapeutic promise in preclinical models (38).

Beyond its direct effects on tumor cells, EMILIN-1 indirectly influences cancer progression by regulating lymphatic function and maintaining inflammatory microenvironmental homeostasis (14). Environmental factors may also influence the expression of EMILIN-1, for instance, smoking has been associated with alterations in EMILIN-1 transcription and DNA methylation patterns, indicating a potential relationship between environmental exposure and cancer development (43).

In conclusion, EMILIN-1 is an important ECM regulator of cancer biology. It exerts tumor-suppressive effects by modulating integrin dependent RAS/ERK signaling and maintaining tumor microenvironmental homeostasis. Loss of EMILIN-1 function, caused by reduced expression, structural degradation or epigenetic modification, facilitates the tumor progression. With regard to prognosis, molecular classification and targeted intervention, EMILIN-1 is an interesting target for future investigations in precision oncology.

## 5 Clinical application potential and future perspectives

With the accumulating evidence supporting the central role of EMILIN-1 in disease pathogenesis, its potential for clinical translation has increasingly been recognized. Two major areas of early translational progress have been biomarker development and targeted therapeutic approaches. Regarding biomarker development, EMILIN-1 expression has been found to be closely associated with disease prognosis in several conditions. In oncology, elevated EMILIN-1 expression in urothelial carcinoma is associated with favorable clinical outcomes and may serve as a prognostic biomarker for patient stratification (44). In hepatology, EMILIN-1 is significantly increased in liver tissues from patients with nonalcoholic steatohepatitis compared with those at earlier disease stages (45). Moreover, in bronchoalveolar lavage fluid from patients with pulmonary fibrosis, there is a positive correlation between the EMILIN-1 levels and pulmonary function parameters (46). These findings support the potential utility of EMILIN-1 as a biomarker for non-invasive biomarker for disease diagnosis, prognosis assessment, and clinical stratification.

Concerning therapeutic strategies, the antifibrotic, tumor suppressive, and anti-inflammatory features of EMILIN-1 render it an attractive target. Restoring EMILIN-1 expression or modulation of its downstream signaling pathways represents a promising therapeutic approach. In animal models of hepatic, pulmonary, and cardiac fibrosis, gene delivery or supplementation with recombinant EMILIN-1 protein to restore EMILIN-1 activity has been shown to effectively attenuate fibrotic progression. Enhancing the interaction between EMILIN-1 and α4β1 integrin in inflammation associated cancers such as colorectal and gastric cancer may inhibit cancer growth and metastasis by decreasing inflammatory cell infiltration and abnormal lymphangiogenesis in the tumor microenvironment (14, 47). Although these preliminary findings provide a foundation for clinical translation, research on EMILIN-1 is still in the early stages between mechanistic investigation and preclinical development. To date, no clinical trials assessing EMILIN-1 based therapeutic interventions have been initiated.

## 6 Discussion

The present review provides a comprehensive overview of the physiological functions and disease-related roles of EMILIN-1. The findings highlight that EMILIN-1 has emerged as a critical regulator of extracellular matrix homeostasis and disease pathogenesis, with increasing potential for clinical translation. However, several key issues remain to be addressed. The interactions between different EMILIN-1 domains are not yet fully understood at the molecular level, and the regulatory elements controlling ligand binding to the gC1q domain require further characterization (4). Furthermore, the mechanisms underlying the functional reversal of EMILIN-1 activity remain poorly understood, including its transition from a tumor suppressive to a tumor promoting role in renal cancer and other malignancies. Future studies should focus on the signaling pathways operating within the tumor microenvironment including the biphasic regulatory effects of TGF-β and identify the key determinants driving this functional transition (25). From a clinical perspective, the diagnostic and prognostic performance of EMILIN-1 as a biomarker requires validation in large-scale studies, while the safety and efficacy of EMILIN-1 targeted therapeutic strategies need further evaluation in preclinical models.

A major challenge in translating EMILIN-1 into a clinical biomarker is that its detection may be affected by tissue injury, inflammation, and extracellular matrix remodeling processes. For example, EMILIN-1 can be cleaved by neutrophil elastase in inflammatory microenvironments, potentially affecting its abundance and biological activity (16). Moreover, the lack of standardized detection protocols remains a significant limitation, as variation in antibody specificity and assay sensitivity may contribute to inconsistent results, a common challenge in the analysis of extracellular matrix proteins (48). In addition, the association between EMILIN-1 expression and disease stage has not yet been systematically validated in large-scale cohorts, limiting its use for accurate disease stratification and clinical decision-making. Current approaches also do not adequately address disease heterogeneity or the context dependent functional reversal of EMILIN-1 activity observed in renal cancer and other cancers (49).

Several directions for future research warrant further investigation. Structural biology techniques could elucidate molecular mechanisms underlying EMILIN-1 interactions with its ligands and provide a foundation for rational therapeutic design (4). The integration of multi omics technologies, including matrisome proteomics, may enable systematic characterization of EMILIN-1 regulatory networks across diseases and facilitate the identification of critical downstream effectors (50). Moreover, clinical studies are required to validate the diagnostic and prognostic value of EMILIN-1 as a biomarker and to optimize EMILIN-1 targeted therapeutic strategies. Further investigation of EMILIN-1 in autoimmune and cardiovascular diseases may expand its clinical relevance and applications.

## 7 Conclusion

EMILIN-1 is a major extracellular matrix molecule that integrates structural and signaling functions, influencing tumor progression, vascular disorders, and chronic inflammatory diseases through integrin mediated signaling, TGF-β/Smad pathways, cell cycle regulation, and lymphangiogenesis. Its effects are highly context dependent, generally conferring tumor suppressive effects, antifibrotic, and anti-inflammatory effects while pro-tumorigenic activity has been observed only in specific cancer types. The molecular mechanisms and translational potential of EMILIN-1 have been increasingly elucidated, although important knowledge gaps remain. Further studies aimed at defining the regulatory mechanisms governing EMILIN-1 expression and stability, as well as strategies to prevent its proteolytic degradation, may facilitate its clinical translation as a diagnostic biomarker and therapeutic target.
